# Preparation of a Silicon/MXene Composite Electrode by a High-Pressure Forming Method and Its Application in Li^+^-Ion Storage

**DOI:** 10.3390/molecules30020297

**Published:** 2025-01-13

**Authors:** Yonghao Liu, Dawei Zhao, Lujia Cong, Yanfeng Han, Mingdi Fu, Xiaoxin Wu, Junkai Zhang

**Affiliations:** 1 Heilongjiang Provincial Key Laboratory of Oilfield Applied Chemistry and Technology, School of Mechatronics Engineering, Daqing Normal University, Daqing 163712, China; yonghaoliu1980@163.com (Y.L.); confidence0001@126.com (D.Z.); conglj378@nenu.edu.cn (L.C.); 2 Key Laboratory of Functional Materials Physics and Chemistry, Ministry of Education, College of Physics, Jilin Normal University, Changchun 130103, China; hyf43411960@outlook.com (Y.H.); a1254012437@163.com (M.F.); xiaoxin.wu@hotmail.com (X.W.); 3 The Joint Laboratory of MXene Materials, Jilin Normal University & Jilin 11 Technology Co., Ltd., Changchun 130103, China

**Keywords:** silicon, MXene, high pressure, lithium ion

## Abstract

The main component of high-capacity silicon-based electrodes is silicon powder, which necessitates intricate processing to minimize volume growth and powder separation while guaranteeing the ideal Si content. This work uses the an situ high-pressure forming approach to create an MXene/*m*-Si/MXene composite electrode, where MXene refers to Ti_3_C_2_T_X_, and *m*-Si denotes two-phase mixed nano-Si particles. The sandwich shape promotes silicon’s volume growth and stops active particles from spreading. The conductive structure of Ti_3_C_2_T_X_ MXene increases the efficiency of charge transfer while reducing internal resistance. After 100 cycles, the composite electrode’s original capacity of 1310.9 mAh g^−1^ at a current density of 0.5 A g^−1^ is maintained at 781.0 mAh g^−1^. These findings lay the foundation for further investigations into Si matrix composite electrodes.

## 1. Introduction

The electrode is one of the most crucial parts of a lithium-ion battery. Silicon is the most promising electrode material for commercial applications. A low electrochemical potential, a high theoretical specific capacity, and an abundance of sources characterize silicon. However, there are certain drawbacks of the silicon electrode as well, such as large volume expansion during charge and discharge, capacity attenuation, and contact at the electrode/electrolyte interface [1,2,3,4,5]. Many efforts have been made by researchers to address these issues. On the one hand, nanocrystallization of silicon materials, the creation of porous structures, and the application of protective layers can reduce the volume expansion of silicon materials and improve their cycle stability and capacity retention rate [6]. On the other hand, by creating a new electrolyte, modifying the interface, and adding alloying components, the interaction between the silicon anode and the electrolyte can be enhanced to enhance battery performance [7,8]. Furthermore, fresh ideas and opportunities for further optimization of silicon anode materials have been presented by a few current research fields and tactics, such as the application of two-dimensional materials, interface engineering, and nanocomposite material design.

A class of metallic carbon (nitrogen) compound nanosheets in two dimensions is called MXene. Many studies are focused on employing MXene as a high-rate electrode material or conductive framework to facilitate energy storage of active materials because of its important properties, which include a large surface ratio, metal-like conductivity, changeable surface functional groups, and a two-dimensional layered structure [9,10,11,12]. It is anticipated that a silicon and MXene composite electrode will offer improved cycle stability and electrochemical performance through sensible design and advancements. First off, MXene exhibits strong mechanical stability and electrical conductivity, which can enhance the conductivity and mechanical stability of the silicon electrode and thus slow down silicon volume change. Second, a stable interface layer can be formed, and the electrode’s cyclic stability can be enhanced by reacting with lithium ions and other electrolyte constituents due to the abundance of surface functional groups present in MXene materials. The electrode’s capacity retention and rate performance can also be enhanced by the silicon and MXene composite electrode’s greater surface area and improved ion diffusion channels. The preparation of silicon and MXene composite electrodes is multifaceted. Direct mixing of silicon nanoparticles with MXene materials is a popular technique, followed by mechanical mixing, solvent treatment, hot pressing, and other techniques to create composite electrodes [13]. An alternative method is to cover the silicon surface with MXene before assembling the composite electrode [14]. Moreover, research has been performed on the formation of silicon/MXene composite electrodes by embedding silicon nanoparticles into the porous structure of MXene [15].

Here, we use a high-pressure technique to create a composite electrode composed of Si nanoparticles and MXene films. The interface resistance could be decreased, and the electron/ion transmission efficiency could be increased by integrating the electrode material with the MXene thin films. This enhanced the battery’s capacity to charge and discharge. High pressure can also be utilized to alter the silicon materials’ structure–activity connection. Under extreme pressure, several isomers of silicon have actually been discovered [16,17,18,19,20]. At room pressure, the diamond cubic (DC-Si, Si-I) structure is the most stable phase of silicon. Si goes through several phase changes in response to pressure. The Si-I, Si-II (*β*-Sn phase), Si-III (BC8, Ia$\overset{-}{3}$), Si-IV (HD-Si, P6_3_/mmc), Si-XII (R8, R$\overset{-}{3}$), and other phases have all been identified between 1 atm and 40 GPa. Of these, Si-III (also known as the cubic structure BC8-Si) is a direct band gap phase that exhibits high voltage and high conductivity. Both energy storage and optoelectronics could be areas of possible application for it. However, due to the constraints of the experimental setup, the large-scale manufacture of BC8-Si has proven challenging in previous research [21,22]. To obtain the BC8-Si phase, DC-Si must typically be subjected to a pressure of 11–14 GPa at room temperature to form the metal *β*-Sn-Si and then decompress from the Si-II phase to control the rate of pressure release, which causes the sample structural changes to follow different sequences. The R8 phase is created by gradually reducing pressure between 10 and 2 GPa. The BC8 phase is then formed by further depression to atmospheric pressure. Nevertheless, this technique necessitates high pressure and a precise pressure release rate control [23].

Considering the difficulties in producing BC8-Si and the feasibility of integrated electrodes, we innovatively synthesized MXene/*m*-Si/MXene composite electrodes in situ using a high-pressure preparation process (where MXene stands for Ti_3_C_2_T_X_, and *m*-Si for two-phase (DC and BC8 phases) mixed nano-Si particles). Firstly, high-pressure shear was applied using the two top anvils of a press with a force of 1400 KN (~1.96 GPa) to synthesize two-phase mixed nanoparticles (*m*-Si) comprising the DC-Si and BC8-Si phases via torsional force (30,000 Nm). Then, by filtering and pressurizing the MXene film, a sandwich-structured MXene/*m*-Si/MXene electrode was created. Lastly, we examined the composite anode’s shape, structure, and electrochemical performance. The special sandwich design reduced silicon volume expansion, prevented particle dispersion, and enhanced the battery cycle and rate performance. These findings offer a significant theoretical and experimental foundation for additional research into this material’s potential applications.

## 2. Results and Discussion

### 2.1. Crystal Phase Analyses

**Figure 1:**
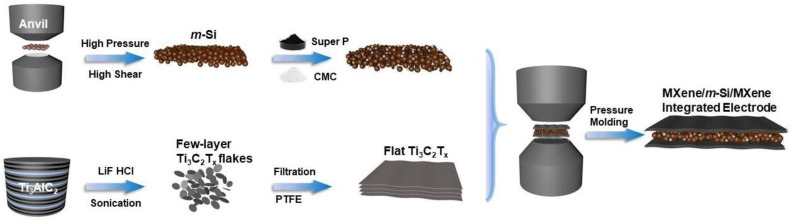
MXene/*m*-Si/MXene composite electrode processing flow chart.

**Figure 2:**
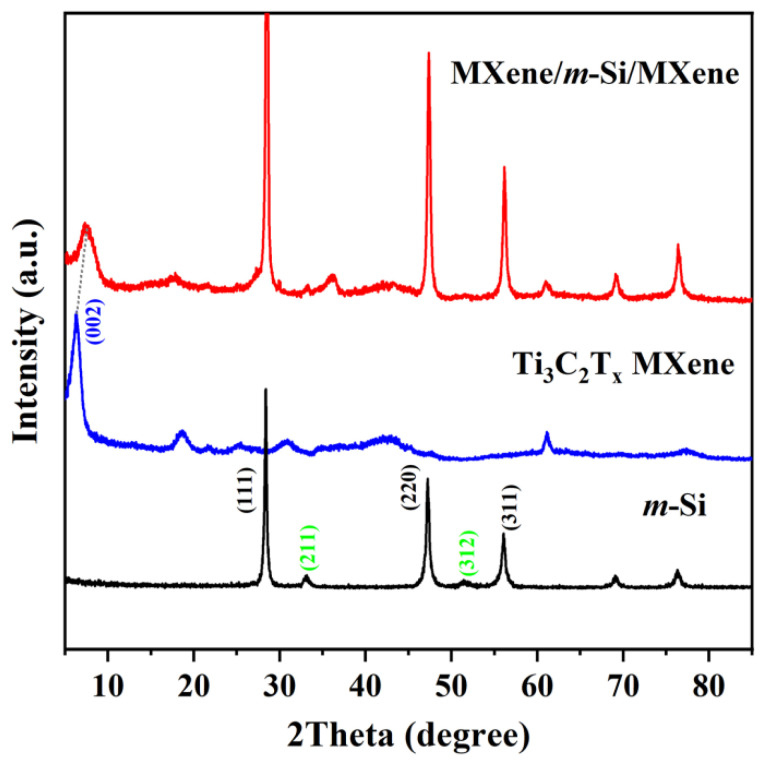
XRD images of *m*-Si particles, Ti_3_C_2_T_X_ MXene thin films, and MXene/*m*-Si/MXene composites.

The characteristic diffraction peak of the pre-etched Ti_3_AlC_2_ sample vanished when we examined the XRD results of Figure S1 in the Supplementary Materials, and the new characteristic diffraction peak of Ti_3_C_2_T_X_ MXene emerged. These findings are in line with earlier findings [9,11]. The effective etching of Ti_3_AlC_2_ into Ti_3_C_2_T_X_ MXene nanosheets with fewer layers was demonstrated (Figure 1). The characteristic peaks of nano-silicon (denoted as *m*-Si) generated by the high-pressure shear technique and Ti_3_C_2_T_X_ MXene concurrently occurred for the MXene/*m*-Si/MXene composites, as shown in Figure 2, when the peaks of the XRD patterns were compared. The DC-Si phase of the face-centered cubic structure is represented by the peaks (111), (220), and (311), whereas the BC8-Si phase of the body-centered cubic structure is represented by the peaks (211) and (312) [24,25]. Using Jade 9.0 software, we calculated the mass fraction of the substance based on the RIR method and found that the content of the newly emerged BC8-Si phase was approximately 9%. Furthermore, we compared the conductivity and carrier concentration of silicon with (*m*-Si) and without (denoted as *p*-Si) the BC8-Si phase, as illustrated in the Table S1. The Hall effect measurements revealed that the resistivity of Si containing both phases, i.e., *m*-Si, was significantly one order of magnitude lower than that of Si without the BC8-Si phase, i.e., *p*-Si. This reduction in resistivity can be attributed to the fact that the carrier concentration in two-phase silicon nanoparticles was nearly an order of magnitude higher. In addition, the shift of the MXene (002) characteristic peak towards higher angles indicated the contraction of the high-pressure substructure, thereby facilitating a more effective control over changes in silicon particle volume. When the upper and lower Ti_3_C_2_T_x_ MXene films encapsulated the middle Si layer, the mutual compression between the layers exerted additional stress on the outer MXene films, leading to a reduction in the interlayer spacing of Ti_3_C_2_T_x_ MXene. This phenomenon was also reflected in the XRD characteristic peaks of the Si layer. It is evident that high-pressure molding techniques were effectively used to create MXene/*m*-Si/MXene composites.

### 2.2. Microstructure and Morphology Analyses

**Figure 3:**
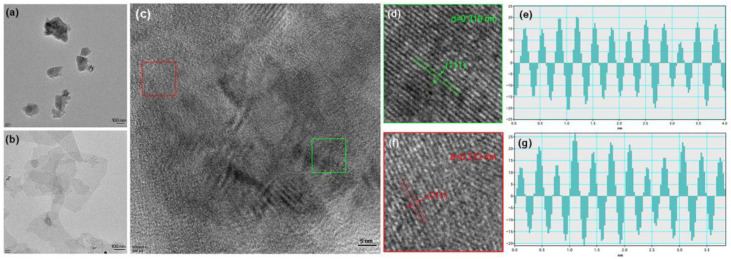
TEM topographical images of *m*-Si (**a**) and few−layer Ti_3_C_2_T_X_ MXene (**b**), HRTEM of *m*-Si particles (**c**), enlarged areas in red and green boxes (**d**,**f**), and corresponding lattice spacing (**e**,**g**) in (**c**).

**Figure 4:**
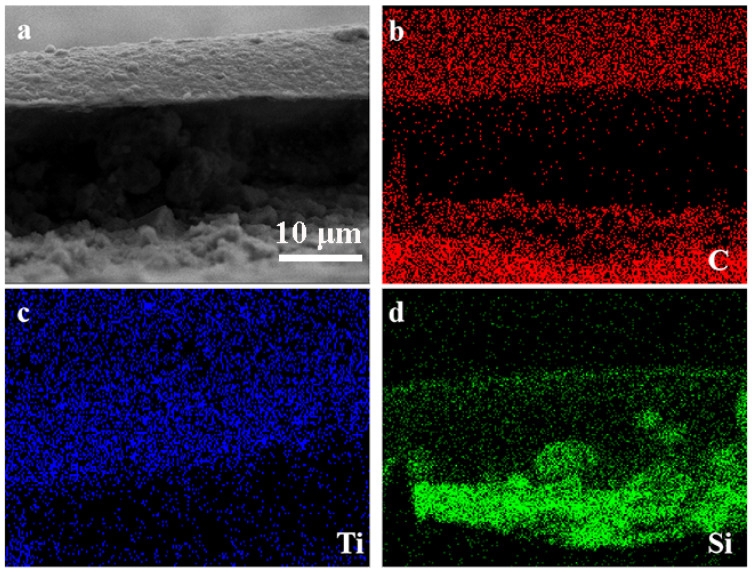
(**a**) Sectional SEM images and (**b**–**d**) corresponding EDS images of MXene/*m*-Si/MXene composite electrode.

The microstructure and morphology of the two distinct materials are characterized in Figure 3a,b prior to high-pressure molding to create MXene/*m*-Si/MXene composites. Additionally, Figure 3c demonstrates that nano-silicon produced by a high-pressure torsion of 360° (axial pressure 1400 KN and torsional force 30,000 Nm) was in a two-phase coexistence state. The crystal face spacing *d* = 0.272 nm corresponding to the peak of BC8-Si (211) is shown in addition to the crystal face corresponding to the peak (111) of the usual face-centered cubic structure. This modification suggests that the Si crystals’ structure was altered. resulting in a tighter arrangement, during the high-pressure shear treatment. The Ti_3_C_2_T_X_ MXene samples displayed the morphology of nanosheets with few layers prior to the production of the composite. The SEM image in Figure 4 illustrates the shape of the composite material, which was created by combining the vacuum filtering process with high-pressure molding. The cross-sectional SEM pictures (Figure 4a) and EDS images (Figure 4b–d) of the MXene/*m*-Si/MXene composite electrode show a sandwich-like structure that might improve battery performance (see electrochemical studies below).

### 2.3. XPS Analysis

**Figure 5:**
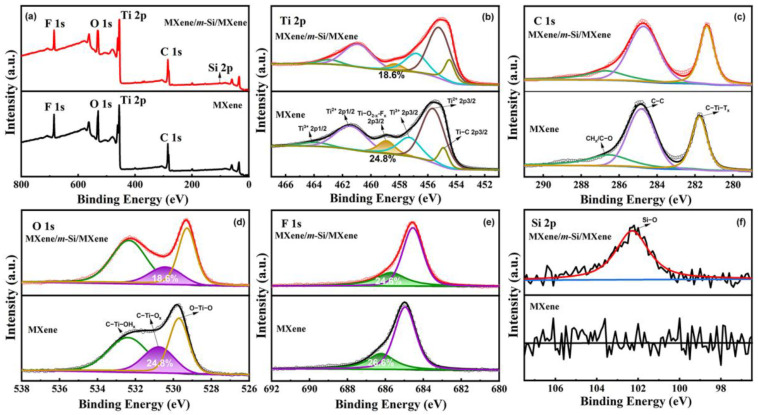
XPS spectra of Ti_3_C_2_T_X_ MXene thin films and the MXene/*m*-Si/MXene composite electrode. (**a**) Total spectrum, (**b**) Ti 2p, (**c**) C 1s, (**d**) O 1s, (**e**) F1s, and (**f**) Si 2p.

There are particular difficulties in utilizing X-ray photoelectron spectroscopy (XPS) to analyze MXenes, and there is currently disagreement over what is responsible for certain XPS characteristics shown in the spectra of materials like Ti_3_C_2_T_x_ [26,27]. Additional analysis of Ti_3_C_2_T_x_ XPS data have been carried out in a number of research studies [26,27,28,29]. By examining the pertinent literature, we improved our knowledge of the composition and surface chemical characteristics of MXene materials in this work. The outcomes demonstrated that the two substances created a sandwich structure concurrently with the formation of a new chemical bond. The combined XPS spectra of the MXene/*m*-Si/MXene composite electrode and the Ti_3_C_2_T_X_ MXene film are displayed in Figure 5a. In agreement with the infrared results of Ti_3_C_2_T_X_ MXene (Figure S2), the energy spectra of the two are essentially consistent, offering direct proof for the presence of C, Ti, O, and F in the sample. The monatomic Al layer in the Ti_3_AlC_2_ MAX phase precursor was selectively etched in LiF and diluted HCl to create Ti_3_C_2_T_X_, so that MXene was in a mixture of F, O, and OH functional groups [9,10,11,12]. Nevertheless, for the MXene/*m*-Si/MXene complex electrode, an XPS signal corresponding to the Si-O bond was also seen at a potential of about 100 eV. After fitting the high-resolution spectra of Ti 2p, six peaks with relative energies of 454.9, 455.7, 457.3, 458.9, 461.5, and 465.0 eV are shown in Figure 5b. They correspond to Ti-C 2p_3/2_, Ti^2+^ 2p_3/2_, Ti^3+^ 2p_3/2_, Ti-O_2-X_-F_X_ 2p_3/2_, Ti^2+^ 2p_1/2_, and Ti^3+^ 2p_1/2_, respectively. Ti atoms in different oxidation states (Ti, Ti^2+^, Ti^3+^) and Ti_3_C_2_F_x_ account for the majority of species. Furthermore, oxidized species were found, such as TiO_2-x_F_x_, in which Ti is linked to both O and F atoms. The C 1s region in Figure 5c was deconvoluted into three separate peaks. The C-Ti-T_x_ bonding is responsible for the most noticeable peak, which is located at 282.0 eV. Graphitic C-C and CH_x_ or C-O species are responsible for the remaining two peaks. Three peaks corresponding to the TiO_2_, C-Ti-Ox, and C-Ti-(OH)_x_ bonds, located at 530.8, 532.4, and 529.7 eV, respectively, were fitted to the O 1s spectra in Figure 5d. Two component peaks of 685.4 and 686.6 eV were fitted to the F1s spectra in Figure 5e. As the composite electrode was successfully prepared, we also noticed that the Ti-O_2-x_-F_x_ (2p_3/2_) XPS peak in MXene became weaker. In addition to creating a stable macroscopic structure, the coupling of Si particles and MXene nanosheets also caused atomic-level changes in the valence state of Ti and the surrounding chemical environment, which is also a useful discovery for the control of the chemical and electronic structures of composite materials. Understanding the general chemical characteristics and stability of composite materials is beneficial in light of this.

### 2.4. Electrochemical Performance

**Figure 6:**
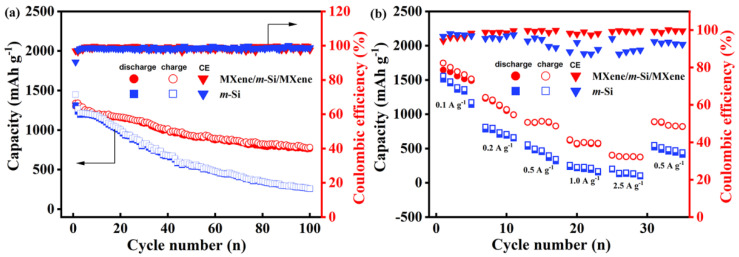
(**a**) Cyclic performance at a current density of 0.5 A g^−1^ and (**b**) rate properties at different current densities of *m*-Si electrode and MXene/*m*-Si/MXene composite electrode.

The electrochemical characteristics of the two electrodes at a current density of 0.5 A g^−1^ are displayed in Figure 6a. The MXene/*m*-Si/MXene composite electrode had an initial capacity of 1310.9 mAh g^−1^. After 10, 50, and 100 cycles, the capacity decreased to 1192.6 mAh g^−1^, 920.5 mAh g^−1^, and 781.0 mAh g^−1^, in that order. The *m*-Si electrode’s initial capacity was 1448.6 mAh g^−1^; after 10, 50, and 100 cycles, it was 1169.7 mAh g^−1^, 542.0 mAh g^−1^, and 258.3 mAh g^−1^, respectively. Additionally, the MXene/*m*-Si/MXene composite electrode exhibited better discharge capacity and cycle stability when paired with the Ti_3_C_2_T_X_ MXene film, whose contribution to capacity was essentially negligible (as shown in Figure S3). The MXene/*m*-Si/MXene composite electrode performed better electrochemically at different current densities, particularly when there was a high rate of charge and discharge, as shown in Figure 6b. The electrode’s specific discharge capacity was measured at 627.4 and 402.0 mAh g^−1^ when the current density was increased to 1.0 A g^−1^ and 2.5 A g^−1^, respectively. The cycle capacity increased rapidly to 891.4 mAh g^−1^ when the current density was changed to 0.5 A g^−1^. The superior specific capacity and rate performance of the MXene/*m*-Si/MXene composite electrode were well demonstrated by this good performance recovery.

**Figure 7:**
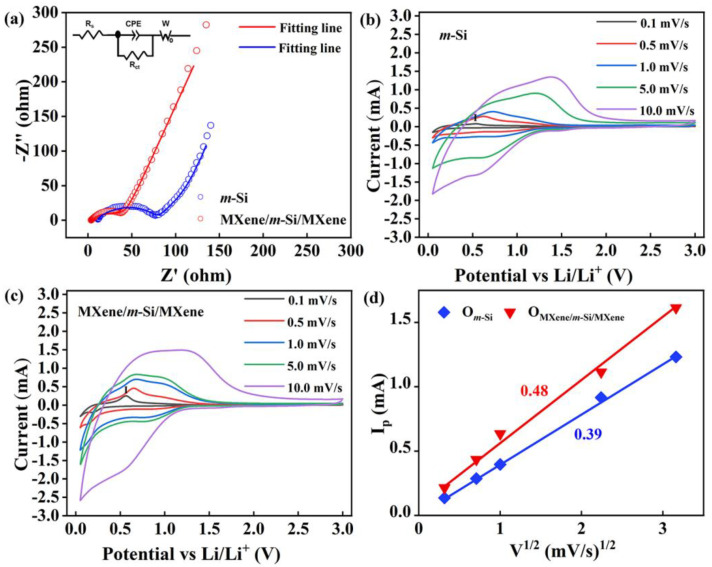
(**a**) Electrochemical impedance spectra (EIS), (**b**,**c**) CV curves at different scan rates, and (**d**) the relationship between peak current (peak I, *I*_p_) and *v*^1/2^ of the MXene/*m*-Si/MXene composite electrode and *m*-Si electrode. Inset in (**a**) is the schematic diagram of the equivalent circuit; therein, *W*_o_ donates the Warburg coefficient.

A Nyquist plot of the Z′/Z″ plane is shown in Figure 7a. The contact resistance (*R*_0_) is represented by the intersection of the high-frequency region with the *x*-axis, and the charge transfer resistance (*R*_ct_) is represented by the semi-arc. The contact resistance *R*_0_ and charge transfer resistance *R*_ct_ values for the *m*-Si and MXene/*m*-Si/MXene-composite electrodes were 11.7 Ω and 67.9 Ω, and 4.1 Ω and 34.9 Ω, respectively. The findings demonstrate that the MXene/*m*-Si/Mxene composite electrode’s performance in charge transfer was superior during the insertion and removal of lithium ions. Moreover, the second-cycle GCD profiles of both electrodes at a current density of 0.2 A g^−1^ were obtained for comparison, as shown in Figure S4. The distinct reaction characteristics of the electrode materials during charge and discharge could be acquired by further analyzing the electrode’s cyclic voltammetry (CV) curves at various scan rates. The CV curves for the *m*-Si and MXene/*m*-Si/MXene-composite electrodes at various scan rates (0.1, 0.5, 1.0, 5.0, and 10.0 mV/s) are displayed in Figure 7b,c. The alloying reaction (Li_x_Si) between silicon and lithium ions is represented by a reduction peak that is seen during the reduction process at 0.20 V [30,31]:

Si + x Li^+^ + x e^−^ → Li_x_Si (1)

The oxidation peaks at 0.30 V and 0.55 V during the oxidation process are ascribed to the Li_x_Si’ dealloying reaction:

Li_x_Si → Si + x Li^+^ + x e^−^ (2)

This procedure demonstrated the silicon-based material’s good reversibility and efficient delithiation [14,32]. The growth of the CV curve area was the result of the anode peak moving towards the high potential and the cathode peak moving towards the low potential, as shown in Figure 7b. This could be brought on by an increase in the electrode’s polarization effect as the scanning rate increased. The fact that all of the CV curves at various scanning rates had comparable forms also suggests that MXene/*m*-Si/MXene composite electrodes work well at different rates. The *m*-Si electrode’s CV curve is displayed in Figure 7b. It is evident from a comparison with Figure 7a that the *m*-Si electrode’s integrated area and peak strength are smaller than those of the composite electrode.

In particular, we computed the peak current (peak I, *I*_p_) of the CV curve for the *m*-Si electrode and the MXene/*m*-Si/MXene complex electrode at various scan rates as a function of *v*^1/2^. As shown in Figure 7d, the cathodic slope for the MXene/*m*-Si/MXene composite electrode (0.48) is relatively higher than that for the *m*-Si electrode (0.39). Accordingly, the Li^+^ ion diffusion coefficients (*D*_Li_^+^) of the two electrodes were calculated based on the Randles–Sevchik Equation (3) [33]:

(3) $$I_{p}=2.69\times 10^{5}An^{3/2}C_{0}D_{Li}^{1/2}v^{1/2}$$

where *n* is the charge transfer number, *A* is the electrode surface area, *C*_0_ is the Li^+^-ion molar concentration, and *D*_Li_^+^ is the Li^+^-ion diffusion coefficient. Compared to the *m*-Si electrode (*D*_Li_^+^~1.65 × 10^−12^ cm^2^/s), the MXene/*m*-Si/MXene composite electrode (*D*_Li_^+^~2.43 × 10^−12^ cm^2^/s) showed a much greater Li^+^-ion diffusion coefficient. Li^+^-ion migration mostly happens when silicon (or Li_x_Si) and the electrolyte come into solid–liquid contact. The increased tension between particles caused by silicon’s volume expansion during charge and discharge cycles improves the solid–solid interactions and impedes lithium-ion migration. Thus, this composite structure improves the movement of lithium ions in comparison to that of electrodes made of pure silicon.

### 2.5. Stability Analyses

**Figure 8:**
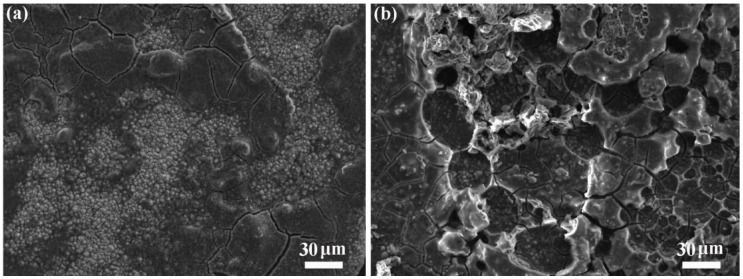
SEM images of (**a**) MXene/*m*-Si/MXene composite electrode and (**b**) *m*-Si electrode at 350× magnification after 100 charge–discharge cycles.

**Figure 9:**
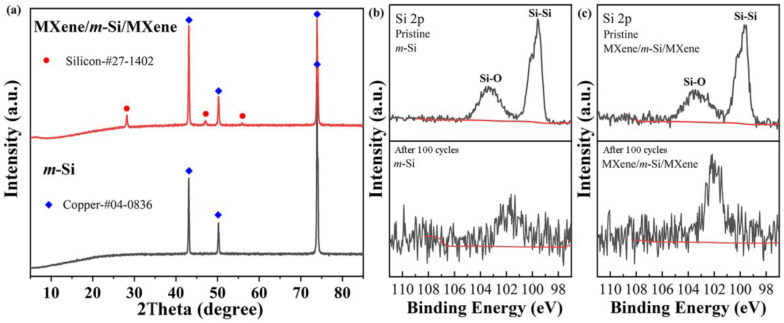
(**a**) XRD patterns of the *m*-Si electrode and MXene/*m*-Si/MXene composite electrode after 100 charge–discharge cycles, (**b**,**c**) XPS spectra of the two electrodes before and after 100 charge–discharge cycles.

The MXene/*m*-Si/MXene composite electrode’s special sandwich structure was primarily responsible for the enhanced electrochemical performance. The construction successfully mitigated the Si particles’ volume expansion, minimizing the impact on the electrode structure (see Figure 8 and Figure S5), by sandwiching the Ti_3_C_2_T_X_ MXene layer between the Si particles and the diaphragm. On the other hand, the intrinsic conductivity of the Si particles remained constant. However, in composite electrodes, the middle *m*-Si layer is tightly encapsulated by highly conductive MXene layers through the application of pressure and temperature. This ensures robust electrical contact between the current collector (i.e., the MXene layer) and the active material. Moreover, Ti_3_C_2_T_X_ MXene, as a conductive medium with abundant active sites on its surface, provides additional conductive pathways. Consequently, the battery’s internal resistance is reduced, and the battery’s energy conversion efficiency is increased to a certain extent. To better elucidate the structural and electronic changes of the active material Si after cycling, we examined the XRD and XPS patterns of both electrodes after 100 charge–discharge cycles. The diffraction peaks of crystalline Si were no longer visible for the m-Si electrode after cycling, as seen in Figure 9a. The main cause of this was the amorphization of the silicon particles and large volume changes resulting in the pulverization of the silicon particles [34]. On the other hand, the diffraction peak of the DC phase was still visible for the composite electrode, even though the distinctive Si diffraction peak of the BC8 phase had vanished. This implies that the MXene coating had a positive impact. Accordingly, the XPS analysis first showed peaks that corresponded to Si-O and Si-. As shown in Figure 9b,c, the Si peak intensities for both electrodes were considerably reduced following cycling [34,35]. Here, we used the Leica U70 ultra-thin slicing mechanism to create semi-thin sections (about 15 μm thick) that exposed the Si layer in order to make it easier to see the Si layer inside the sandwich structure. The Si electrode material on the copper foil was then directly observed.

## 3. Experimental Description

### 3.1. Preparation of the MXene/m-Si/MXene Composite Electrode and m-Si Electrode

Firstly, Ti_3_C_2_T_X_ MXene nanosheets at a concentration of 10 mg/mL were prepared using a solution containing LiF and 6 M HCl. Vacuum filtering was then used to prepare thin films of Ti_3_C_2_T_X_ MXene. The thickness of each film was within the range of 7–10 μm. On the other hand, the two-phase mixed nanoparticles (noted *m*-Si) powder was pre-produced by high-pressure shear (axial pressure 1400 KN and torsional force 30,000 Nm, rotating by 360 degrees) of pristine micron silicon (noted *p*-Si). Therein, *m*-Si and *p*-Si exhibited significant differences in electrochemical performance (see Supplementary Materials Figure S6). Subsequently, three layers of material (two Ti_3_C_2_T_X_ MXene films and a middle *m*-Si layer) stacked in sequence were placed on the press lower anvil with a diameter of 300 mm, and in situ baking was conducted for 60 min at 60 °C, also maintaining the pressure of 300 KN. Finally, a 12 mm diameter wafer was produced using a tablet press. The *m*-Si electrode was created by applying active material slurries to the surface of a copper foil that was 9 μm thick. Digital images are included in the Supporting Information Figure S7 to show the MXene/*m*-Si/MXene composite electrode’s and the *m*-Si electrode’s thickness. The Supplementary Materials contains a description of the preparation procedure in detail.

### 3.2. Material Characterization

An X-ray diffractometer (XRD, JEOL DY01660, JEOL (Beijing) Co., Ltd., Beijing, China) using Cu *K*α radiation (40 kV, 200 mA, *λ* = 1.5418 Å), a transmission electron microscope (TEM, JEOL 2100, JEOL (Beijing) Co., Ltd., Beijing, China), a scanning electron microscope (SEM, JEOL JSM-7800F, JEOL (Beijing) Co., Ltd., Beijing, China), an energy-dispersive X-ray spectrometer (EDS, Oxford UltimMax100, Oxford Instrument Technology (Shanghai) Co., Ltd., Shanghai, China), an X-ray photoelectron spectroscope (XPS, Thermo Scientific Escalab250Xi, Thermo Fisher Technology (China) Co., Ltd., Shanghai, China) employed with a monochromatic Al-*K*α X-ray source, and a Fourier transform infrared spectrometer (FTIR, PerkinElmer Frontier, Perkinelmer Instruments (Shanghai) Co., Ltd., Shanghai, China) using the transmission mode were used to methodically examine the prepared samples. See Supplementary Materilas for specific experimental parameters.

### 3.3. Cell Fabrication and Electrochemical Evaluation

The active material loading for the two electrodes indicated above was roughly 0.8–1.0 mg/cm^2^. CR2032 coin cells were used to assemble half cells, with lithium metal serving as the counter electrode and a polypropylene separator. In order to create the electrolyte, LiPF_6_ was dissolved in a 1:1:1 volume ratio of ethylene carbonate (EC), diethyl carbonate (DEC), and ethyl methyl carbonate (EMC). The two electrodes’ electrochemical characteristics were assessed using the apparatus and parameter configurations outlined in the Supporting Information.

## 4. Conclusions

By incorporating Ti_3_C_2_T_X_ MXene and employing the high-pressure forming technique, a sandwich-structured composite electrode comprising MXene/*m*-Si/MXene was successfully fabricated in this study. Its special geometric structure efficiently mitigates the volume expansion of Si particles, lowers Si loss, and creates a good conducting network, all of which enhance the electrode’s capacity and cycle performance. The development of high-performance lithium-ion batteries will greatly benefit from this. The present study clarifies the process of preparing Si matrix composite electrodes, providing a crucial experimental foundation for future investigations into the potential applications of this material.

## Data Availability

Data are contained within the article and Supplementary Materials.

## Supplementary Materials

The following supporting information can be downloaded at: https://www.mdpi.com/article/10.3390/molecules30020297/s1, Detailed experimental process; Figure S1: XRD patterns of MAX phase Ti3AlC2 and few-layer Ti3C2TX MXene nanosheets; Table S1: Carrier transport parameters determined by Hall effect measurements on p-Si and m-Si samples, where the powders were cold pressed at 210 MPa into thin discs; Figure S2: Infrared spectrum of few-layer Ti3C2TX MXene nanosheet; Figure S3: The cycle performance of Ti3C2TX MXene film at 1 C (1 C = 100 mA g^−1^); Figure S4: The 2nd cycle galvanostatic charge/discharge of m-Si and MXene/m-Si/MXene electrodes at a current density of 0.2 A g^−1^; Figure S5: SEM images of (a) MXene/m-Si/MXene composites electrode and (b) m-Si electrode at 750x magnification after 100 charge-discharge cycles; Figure S6: (a) Electrochemical impedance spectra (EIS), (b) The 2nd galvanostatic charge/discharge curves at a current density of 0.2 A g^−1^ and (c) Cyclic performance at a current density of 0.5 A g^−1^ of p-Si and m-Si electrodes; Figure S7: Digital photographs showing the thickness of the m-Si electrode (a) and the MXene/m-Si/MXene composite electrode (b), respectively.
