# Supplementary material for: Preparation of a Silicon/MXene Composite Electrode by a High-Pressure Forming Method and Its Application in Li+-Ion Storage
Source: Molecules. 2025 Jan 13;30(2):297. doi: 10.3390/molecules30020297 (PMC11767502; doi:10.3390/molecules30020297)
Supplement: Supplementary file 1 [file molecules-30-00297-s001.zip › molecules-3364355-supplementary.pdf]

Preparation of silicon/MXene composite electrode by high-pressure forming method and its application in Li^+ ions storage

(1) The preparation of the $Ti_3C_2T_x$ MXene thin film

The preparation of the $Ti_3C_2T_x$ MXene thin film involved the following steps. Initially, 10 g of Ti_3AlC_2 powders (99.8% weight percentage, supplied by 11 Technology Co., Ltd.) were submerged in approximately 100 mL of a solution containing LiF and 6 M HCl at 30 °C. The suspension was then stirred at 700 rpm for 24 hours. Following this, the suspension was repeatedly washed with deionized water. To facilitate drying at room temperature, the cleaned powder was subjected to vacuum filtration. Subsequently, 0.3 g of the product was dispersed in 150 mL of degassed water. After 20 minutes of ultrasonication under flowing Ar, the mixture was slowly centrifuged to obtain a highly purified $Ti_3C_2T_x$ flake colloidal solution.

Subsequently, ultrasonic treatment was employed to disperse the $Ti_3C_2T_x$ flakes in 10 mL of fresh deionized (DI) water. A thin layer of $Ti_3C_2T_x$ MXene solution was filtered at a flow rate of 10 L/min using a SHZ-D(III) circulating water vacuum pump. To ensure complete separation of the liquid, a filtration membrane with a diameter of 50 mm and pore size of 0.2 μm was utilized. After removing the filter membrane, the sample was left to dry. The $Ti_3C_2T_x$ MXene film was carefully peeled off from the filter membrane once it was fully separated. The thickness of each film was measured using a spiral micrometer, yielding values within the range of 7–10 microns.

(2) The preparation of the two-phase mixed nanoparticles (m-Si)

At room temperature, 500 mg of silicon particles (with an average size of 25 μm) are placed on the anvil surface, using a hydraulic press as the power source. Two parallel cemented carbide blocks, each with a diameter of 30 mm, serve as the anvils. The axial pressure is set to 1400 KN, and the upper and lower anvils gradually come into contact under oil pressure drive. Pressure is incrementally increased to the

predetermined level through squeezing. While maintaining this pressure, the lower anvil is twisted using a hydraulic wrench, applying a torsion force of 21,000 Nm to rotate the anvil one full turn (360 degrees). To synthesize mixed-phase nano-silicon, the applied pressure is held constant for 20 minutes before being fully released.

(3) The preparation of MXene/m-Si/MXene composite electrode and m-Si electrode

The active ingredient was *m*-Si powder. For the MXene/*m*-Si/MXene composite electrode, super P (TIMCAL) and carboxymethyl cellulose (CMC) (DS=0.7, M_w = 90000, Aldrich) were added as conductive agent and binder, respectively. A 30 mm diameter MXene film was coated with a mixture comprising 80 wt% active m-Si material, 12 wt% super P, and 8 wt% CMC, which was then placed on the lower anvil. Subsequently, a second piece of MXene film was positioned on top. The device featured heating plates beneath the two anvils, transferring heat to the sample via the anvils. A K-type thermocouple was placed in the area of the anvil closest to the sample for temperature monitoring. The preset pressure was set at 300 KN, and the temperature was increased to 60 °C at a rate of 10 °C per minute. After maintaining the pressure and temperature for 60 minutes, both were simultaneously released. Finally, a 12 mm diameter wafer was produced using a tablet press. For the *m*-Si electrode, working electrodes were fabricated by coating slurries containing active materials onto the surface of 9 μm thick copper foil using a coater with a 40 μm gap. The electrodes were dried in a vacuum drying oven at 80 °C for 12 hours under vacuum conditions. A 12 mm diameter wafer was also produced using a tablet press.

(4) Material Characterization

An X-ray diffractometer (XRD, JEOL DY01660) was employed to identify the phase composition. The analysis was conducted using Cu Kα radiation (40 kV, 200 mA, λ = 1.5418 Å) with a scanning range of 5° to 85° and a step size of 0.02°. High-resolution imaging was performed using a transmission electron microscope (TEM, JEOL 2100F) equipped with a field emission gun, operating at 200 kV and

featuring a point resolution of 0.19 nm. The chemical composition and morphology of the samples were characterized using a scanning electron microscope (SEM, JEOL JSM-7800F) coupled with EDS (Oxford UltimMax100). X-ray photoelectron spectroscopy (XPS) was utilized to characterize the chemical states of the samples. Specifically, a Thermo Scientific Escalab250Xi instrument was employed with a monochromatic Al-Kα X-ray source (hv = 1486.8 eV) featuring a spot size of 400 μm to irradiate the sample surface. The operating conditions were as follows: anode voltage of 15 kV and filament current of 10 mA. Full-spectrum scans were conducted over an energy range of 150 eV with a step size of 1 eV, while high-resolution scans were performed over an energy range of 50 eV with a step size of 0.1 eV. At least five cycles of signal accumulation were conducted for each element to ensure data reliability. Binding energy calibration was performed using the C1s peak at 284.8 eV as the reference. Prior to analysis, the samples were vacuum-dried under argon protection to prevent environmental contamination. The infrared absorption spectra of the functional groups on the surface of the sample were obtained using the transmission mode of FTIR (PerkinElmer Frontier).

(5) Electrochemical Measurements

For the two aforementioned electrodes, the loading of active materials was approximately 0.8-1.0 mg/cm^2, which is within the commonly accepted range of 0.7-2.0 mg/cm^2. Half cells were assembled using CR2032 coin cells, with a polypropylene separator and lithium metal as the counter electrode. The electrolyte was prepared by dissolving $LiPF_6$ in a mixture of ethylene carbonate (EC), diethyl carbonate (DEC), and ethyl methyl carbonate (EMC) at a volume ratio of 1:1:1. Cyclic voltammetry (CV) curves were obtained on an electrochemical workstation (Shanghai Chenhua Instruments Company, CHI670E) at scan rates of 0.1, 0.5, 1, 5, and 10 mV/s within the voltage window of 0.01-3 V. Electrochemical impedance spectroscopy (EIS) measurements were conducted on the same workstation over a frequency range from 100 kHz to 100 mHz with an amplitude voltage of 5 mV. The

cycling stability and rate performance of the materials were evaluated using a battery test system (Shenzhen Neware Electronics Co., Ltd., CT-ZWJ-4S-T-1U) under conditions of a cycling rate of 0.5 A g^{-1}, rate capabilities of 0.1, 0.2, 0.5, 1.0, and 2.5 A g^{-1}, within the voltage range of 0.01-3 V, and at a temperature of 27 °C.

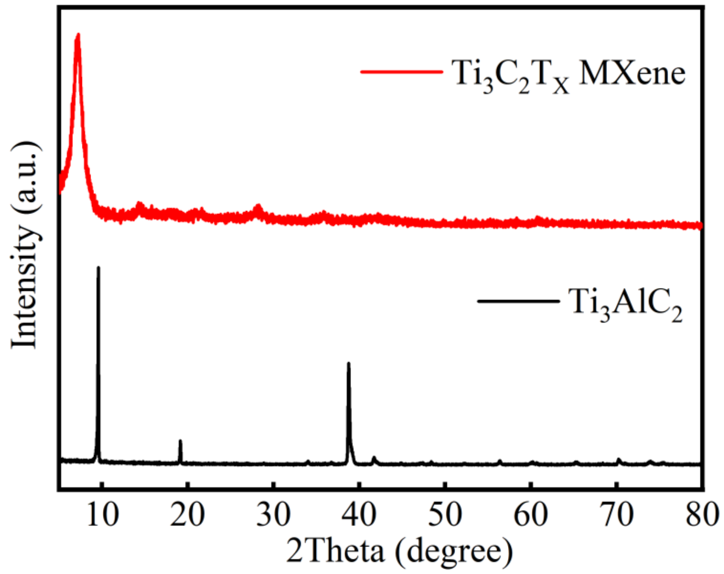

Figure S1. XRD patterns of MAX phase Ti_3AlC_2 and few-layer $Ti_3C_2T_X$ MXene nanosheets.

Table S1. Carrier transport parameters determined by Hall effect measurements on *p*-Si and *m*-Si samples, where the powders were cold pressed at 210 MPa into thin discs.

Sample	Field [G]	Resistivity [ohm·cm]	Hall Coefficient [cm^3/C]	Type	Carrier Density [1/cm^3]	Hall Mobility [cm^2/(VS)]
m-Si	3000	2.434	-5.374×10^2	n	1.161×10^{16}	4.127×10^2
p-Si	3000	15.99	-5.191×10^3	n	1.203×10^{15}	3.986×10^2

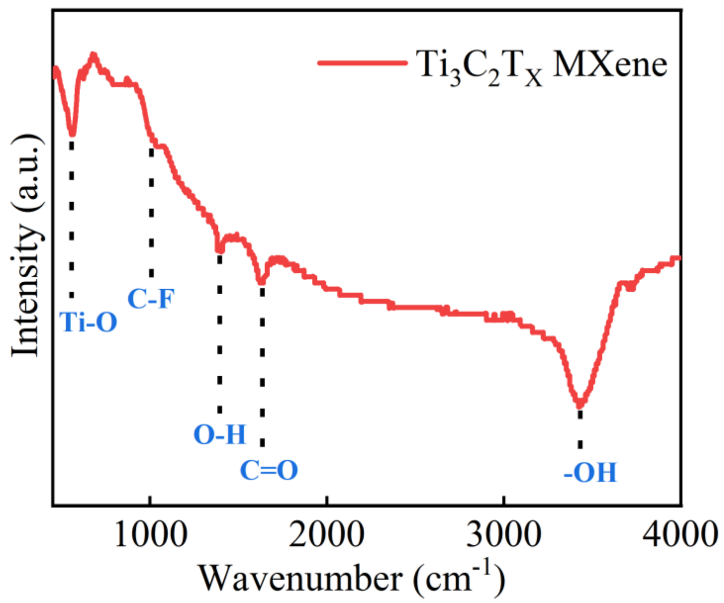

Figure S2. Infrared spectrum of few-layer $Ti_3C_2T_X$ MXene nanosheet.

The defining peak of the Ti-O functional group, which is around 600 cm^{-1}, is visible in Figure S2. This suggests that the sample contains a chemical connection between the atoms of titanium and oxygen. Furthermore, at roughly 1084 cm^{-1}, the distinctive peak of the C-F functional group is seen. This suggests that the sample contains C-F linkages or other fluorinated chemicals. Furthermore, the typical peak of the O-H functional group, which peaks at approximately 1400 cm^{-1}, is clearly visible. This suggests that the sample contains hydroxyl (-OH) functional groups. Furthermore, at roughly 1634 cm^{-1}, the distinctive peak of the C=O functional group is visible. This suggests that the sample has a carbon-oxygen C=O. Lastly, at roughly 3436 cm^{-1}, the distinctive peak of the -OH functional group is visible. This provides additional evidence that the material contains -OH functional groups. We can infer from the aforementioned data that the sample contains a variety of functional groups, including Ti-O, C-F, O-H, C=O, and -OH.

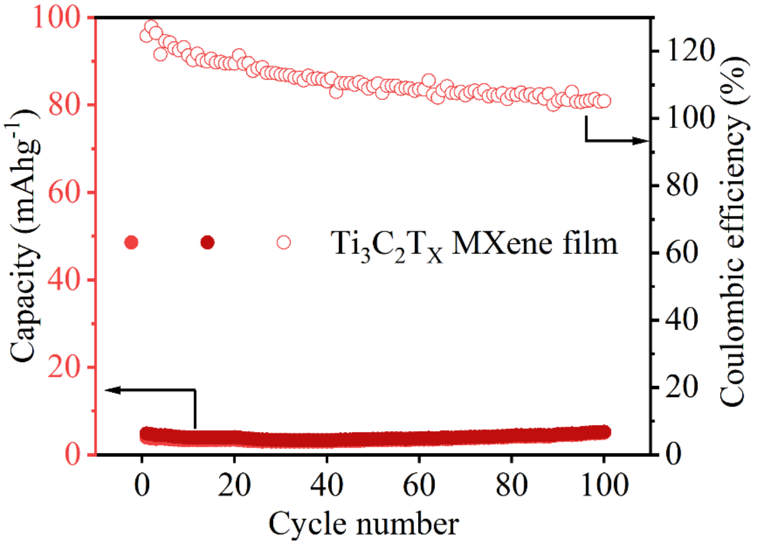

Figure S3. The cycle performance of $Ti_3C_2T_X$ MXene film at 1 C (1 C = 100 mA g^{-1}).

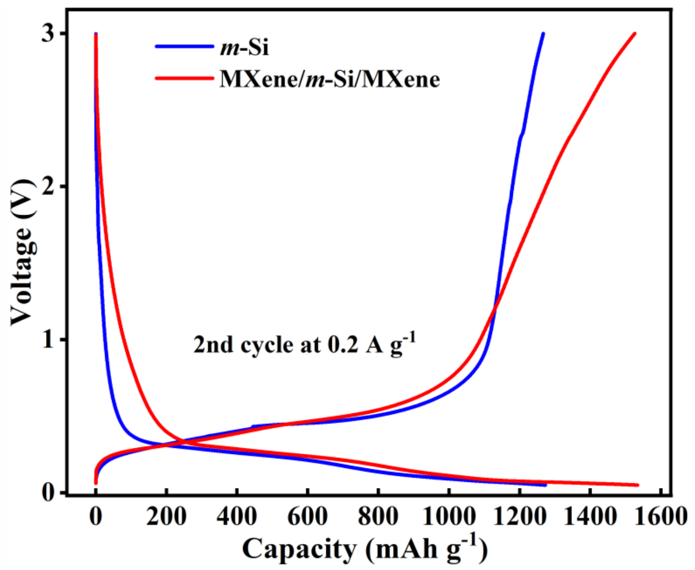

Figure S4. The 2nd cycle galvanostatic charge/discharge of *m*-Si and MXene/*m*-Si/MXene electrodes at a current density of 0.2 A g^{-1}.

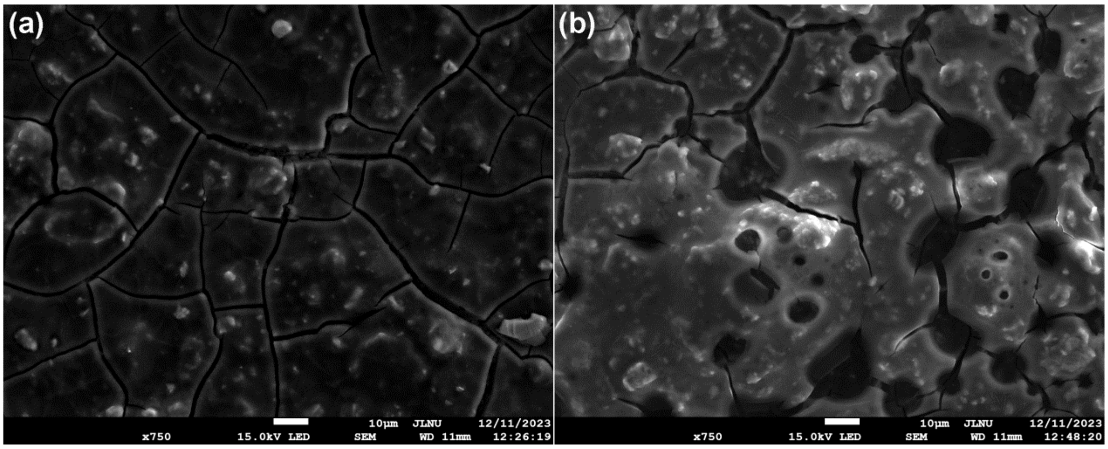

Figure S5. SEM images of (a) MXene/*m*-Si/MXene composites electrode and (b) *m*-Si electrode at 750x magnification after 100 charge-discharge cycles.

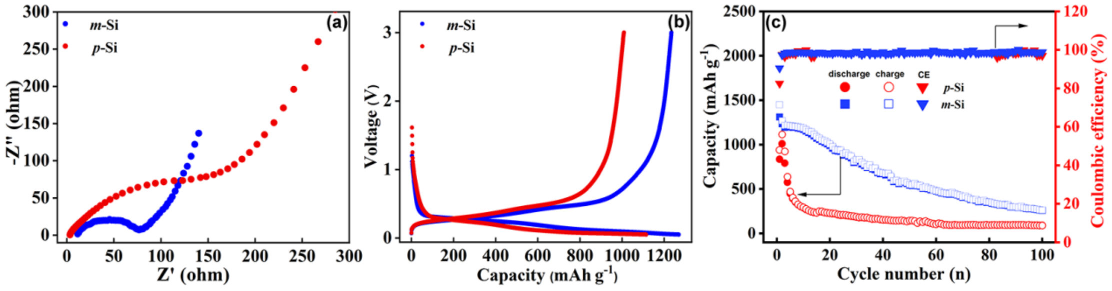

Figure S6. (a) Electrochemical impedance spectra (EIS), (b) The 2nd galvanostatic charge/discharge curves at a current density of 0.2 A g^{-1} and (c) Cyclic performance at a current density of 0.5 A g^{-1} of *p*-Si and *m*-Si electrodes.

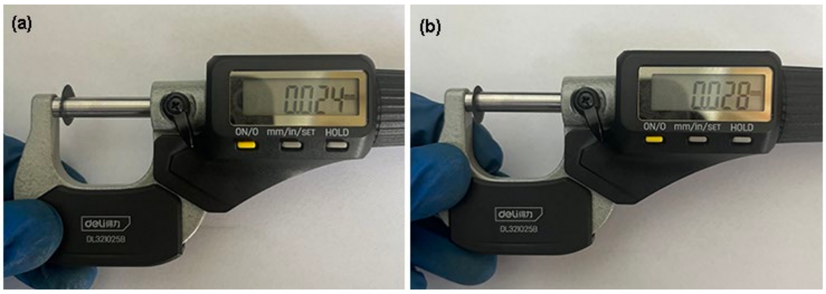

Figure S7. Digital photographs showing the thickness of the *m*-Si electrode (a) and the MXene/*m*-Si/MXene composite electrode (b), respectively.
